# High endemicity of *Clonorchis sinensis* infection in Binyang County, southern China

**DOI:** 10.1371/journal.pntd.0008540

**Published:** 2020-08-10

**Authors:** Junling Sun, Hualei Xin, Zhihua Jiang, Menbao Qian, Kaixia Duan, Yingdan Chen, Shizhu Li, Wei Li, Shiyong Huang, Xiaoqin Gan, Yichao Yang, Zhongjie Li

**Affiliations:** 1 Division of Infectious Disease, Key Laboratory of Surveillance and Early Warning on Infectious Disease, Chinese Centre for Disease Control and Prevention, Beijing, China; 2 Division of Infectious Disease, Qingdao City Centre for Disease Control and Prevention, Qingdao, China; 3 Guangxi Centre for Disease Control and Prevention, Nanning, China; 4 National Institute of Parasitic Diseases, Chinese Centre for Disease Control and Prevention; WHO Collaborative Centre for Malaria, Schistosomiasis and Filariasis; Key Laboratory of Parasite and Vector Biology, Ministry of Health, Shanghai, China; 5 Malaria Control Department, Yunnan Institute of Parasitic Diseases, Yunnan, China; 6 Parasite Disease Control Department, Binyang Centre for Disease Control and Prevention, Nanning, China; 7 Director, Binyang Centre for Disease Control and Prevention, Nanning, China; 8 Deputy Director, Binyang Centre for Disease Control and Prevention, Nanning, China; Istituto Superiore di Sanità, ITALY

## Abstract

High-intensity clonorchiasis infection is associated with serious outcomes, including cancer. Understanding the infection intensity of *Clonorchis sinensis* and its risk factors in local endemic regions could facilitate effective control measures. In a county located in a highly endemic area in Guangxi Province, P. R. China, local residents were randomly enrolled in the study; helminth egg examinations were performed with the Kato-Katz method, and the intensity of infection was identified as mild, moderate or heavy. Knowledge, attitudes, and high-risk behaviours were investigated among those infected with *Clonorchis sinensis*. A total of 2521 local residents participated in this study, and the *Clonorchis sinensis*-positive proportion was 28.9% (728 persons). Among the infected persons, the percentages of mild, moderate and heavy infections were 66.2%, 28.4% and 5.4%, respectively. Males experienced a higher proportion of moderate and heavy infections (37.5%) than females (18.1%) (p<0.05). The highest infection proportion among the different levels of infection intensity was identified among persons aged 30–59 years (15.7% for moderate and heavy infections). Among the 509 persons who reported eating raw fish, 302 persons (59.3%) had eaten raw fresh fish for more than 10 years, and 131 (25.7%) persons ate raw fish ≥12 times a year. Multivariate logistic regression revealed that eating raw fish 12–50 times in the last year (adjusted odds ratio [aOR] = 1.74, 95%CI: 1.09–2.80) and eating raw fish >50 times in the last year (aOR = 2.89, 95%CI: 1.20–7.50) were risk factors for high-intensity infections (moderate and heavy). The overall infection proportion was high in the study area, with a large group of residents experiencing high-intensity infections. High frequency of raw fish consumption was associated with high-intensity infections. Intervention strategies targeting people with a high frequency of raw fish consumption should be implemented to reduce the probability of severe consequences.

## Author summary

Clonorchiasis, which is caused by *Clonorchis sinensis*, is one of the most endemic parasite diseases in China. Humans get infected by eating the raw or undercooked freshwater fish that contain the nymphal stage of *Clonorchis sinensis*. There are about 15 million cases globally with 85% of those in China. Two major endemic areas were found in China, including Guangdong and Guangxi Province in the south part of China, and Heilongjiang, Jilin and Liaoning Province in the north part of China. In this study, by analysing the dataset from one county in Guangxi Province, we found the overall proportion of infection was high, with one third of infection persons were classified as high intensity of infection. Male and persons aged 30–59 years developed higher burden of infection. Besides, persons who eat high frequency of raw fish developed high intensity of infection. Health education on the high risk populations should be strengthened to change the diet custom. Other measure such as examination of freshwater fish in the market should also be implemented.

## Introduction

*Clonorchis sinensis (C*. *sinensis)*, the oriental liver fluke, is an important fish-borne zoonosis. Adult *Clonorchis sinensis*, which are located in the livers of various mammals, including humans, produce eggs that are passed into the intestine. Most of the parasites live in bile ducts, gallbladder and liver parenchyma, causing liver and biliary diseases. Human beings become infected by ingesting raw or undercooked fish that contain the metacercariae of liver flukes [1–5].

Globally, the distribution range of *C*. *sinensis* mainly consists of two epidemic zones. The first zone includes south-eastern China and the northern area of Vietnam, and the second zone covers north-eastern China, the Republic of Korea, part of Russia and probably the Democratic People’s Republic of Korea [5]. The conservative estimate of the population infected with *C*. *sinensis* reached 15 million in 2004 globally, with over 85% of the estimated population in China [5]. There are two major endemic regions in China, i.e., south-eastern China, including Guangdong and Guangxi with prevalence rates of 16.42% and 9.76%, respectively, and north-eastern China, including Heilongjiang, Jilin and Liaoning with prevalence rates of 4.73%, 2.90% and 0.80%, respectively, between 2002–2004 [6–9]. According to the results of three large-scale surveys of clonorchiasis carried out in mainland China, the prevalence of clonorchiasis increased from 0.37% between 1988 and 1992 to 0.58% between 2002 and 2004, which indicated that the population infected with clonorchiasis increased from 4.70 million to 12.49 million people [8–10].

Symptoms caused by clonorchiasis are directly proportional to worm burden [1,6,11]. Hence, patients with low infection intensity are often asymptomatic or show only mild symptoms, whereas patients with high infection intensity often show unspecific symptoms, such as asthenia, nausea, indigestion, headache or abdominal pain, especially in the right upper quadrant [6,12]. In addition, developmental retardation has been reported in children with heavy infection intensity. These children often present with inappetence, diarrhoea, malnutrition, anaemia and hepatomegaly [13]. Cholelithiasis is one of the most frequent complications of such infection. Previous studies discovered that a high worm burden and narrowing of the bile ducts, which might result from the accumulation of worms, cause obstruction, sequential bile stagnation, and bile pigment deposition, which may give rise to the formation of stones in bile ducts with eggs or dead worms as nuclei [14–17]. Additionally, *C*. *sinensis* infection is now widely acknowledged to be associated with cholangiocarcinoma, i.e., bile duct cancer [18,19]. As evidence accrued, *C*. *sinensis* and *Opisthorchis viverrini* were classified in 2009 as definite carcinogens (group 1) by the International Agency for Research on Cancer [18,19]. Systematic reviews and meta-analyses revealed pooled odds ratios (ORs) for *C*. *sinensis* infection and cholangiocarcinoma ranging between 4.5 and 6.1 [5,20,21]. Additionally, in a study focusing on the relationship between liver flukes and cholangiocarcinoma, Odds Ratios (OR) were significantly associated with higher infection intensities [22].

In this study, we aim to explore the epidemiological characteristics of clonorchiasis in local endemic areas, with an emphasis on understanding the distribution of infection intensity and risk factors of high intensity, which can contribute to the effective and sustainable development of intervention measures.

## Methods

### Study site

**Fig 1:**
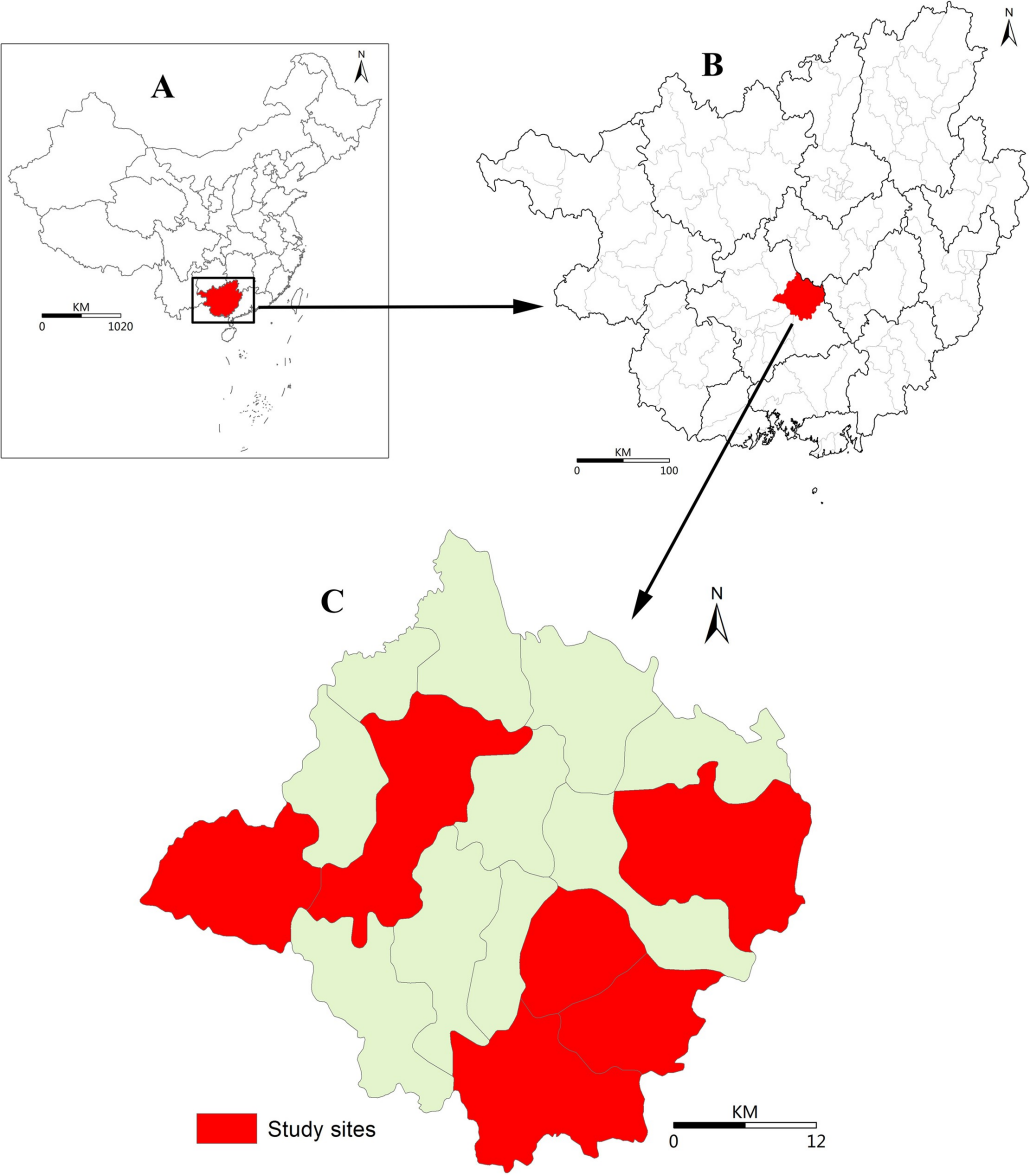
Geographic Location of Study Sites in Binyang County, Guangxi Autonomous Region, China. **A**. Geographic location of the Guangxi Autonomous Region in China. **B**. Location of Binyang County in the Guangxi Autonomous Region. **c**. Location of the study site in Binyang County [24].

The study was carried out in Binyang County, Nanning City, Guangxi Autonomous Region, P.R. China (Fig 1). According to previous studies, clonorchiasis in Guangxi is hyper-epidemic in the Pearl River Basin [23], with 64% of total counties encompassing hyper-endemic zones (infection proportion >10%) [23]. Binyang County is located at the Pearl River Basin, where has 16 towns and a population of approximately 1.1 million. The annual discretionary income per capita reached 33095 CNY for urban citizens and 14038 CNY for rural citizens in 2018.

### Study design and data collection

Among all 16 towns of Binyang County, 7 towns were identified as the survey areas by using a random sampling method, and two villages from each town were further selected. In each survey village, 200 residents were randomly enrolled in the survey. The same questionnaire was used to collect the demographic information, including age, sex, education and occupation, of all enrolled study participants. Furthermore, one stool sample was collected from each participant. Experienced and trained public health staff prepared three Kato-Katz thick smears for each sample and examined them for eggs under a light microscope [25,26], and each smear was examined for different people. Ten stool samples were chosen for each study village (14 for overall) to be re-examined by professional staff in the high level of CDC to conduct quality control. *C*. *sinensis* infection was defined based on the identification of *C*. *sinensis* eggs in stool specimens [25,26].

After the faecal examination, participants who were confirmed to be infected with *C*. *sinensis* were asked to complete the structured questionnaire with trained investigators. The questionnaire contained four parts: demographic characteristics, previous illness history, knowledge and attitude towards clonorchiasis, and risk behaviours related to *C*. *sinensis* infection. In the demographic part, sex, age, occupation and education were recorded. Four questions related to knowledge and attitudes (e.g., “Do you know the transmission route” and “Do you know the most serious consequences caused by clonorchiasis?”) were asked. Other questions about raw fish consumption, such as the duration, frequency, quantity and location of raw fish consumption, were recorded in the risk behaviours part. Data used in our manuscript was based on the *C*. *sinensis* re-infection program conducted in Guangxi Province, China. The main purpose of this program was to explore the re-infection rate and risk factors that influence the occurrence of *C*. *sinensis* among cured clonorchiasis patients. The first step of this program was to screen clonorchiasis patients, then cured them, and followed up them to identify the re-infection situation. Therefore, the program was focused on the infected people, and the structured questionnaire was only used in infected persons. For the negative persons, only basic demographic information was collected.

### Data analysis

The data were double-entered and cross-checked in EpiDate 3.1 software (http://www.epidata.dk/). Analysis was performed in R Studio software (Version 1.0.153, Inc.). The overall infection proportion was computed by dividing the number of infected persons by the total number of survey participants, and the proportion was standardized by using different age groups of the local population (in 2017). The population data used to calculate the standardized proportion were obtained from the Statistics Bureau of Nanning District. The number of eggs per gram of faeces (EPG) was calculated by multiplying the egg count of every smear by 24 and then computing the average of three smears. The intensity of infection was categorized as mild infection (EPG 1–999), moderate infection (EPG 1000–9999) or heavy infection (EPG ≥10000) [20,27].

The overall proportions of mild, moderate and heavy infections were calculated by dividing the number of mild, moderate and heavy infections by the total number of infections. Moreover, the infection proportion and the proportions of the three types of infection intensity (mild infection, moderate infection and heavy infection) for different population groups (by sex, age, occupation and education level) were calculated and compared. Age was transformed into five categories (15, 15–29, 30–44, 45–59, and 59 years).

To understand raw fish consumption behaviour in the study subjects, we calculated and compared the duration (0–5 years, 6–10 years and >10 years), frequency (0–11 times a year, 12–50 times a year and >50 times a year) and quantity (0–9 slices, 10–19 slices and ≥20 slices per serving of raw fish) of raw fish consumption for the overall population and different population groups; the use of anti-parasitics after eating raw fish; and the main location of raw fish consumption. Univariate and multivariate logistic regression models were used to explore the relationship between raw fish consumption behaviour and high-intensity infection (moderate and heavy infections).

Pearson’s χ^2^ test was applied to assess the association between the categorical variables. Statistical significance was determined at a *p*-value of 0.05.

This study was approved by the ethics committees of the Chinese Centre for Disease Control and Prevention (Approval Notice no: 201617). The objectives, procedures and potential risks were orally explained to all participants. A written consent form was also obtained from each participant with his or her own signature or the signature of a proxy, including being administrated the praziquantel to treat clonorchiasis as a medicine with off-label use.

## Results

### Basic characteristics of the study subjects

A total of 2521 subjects (response rate: 90.0%, 2521/2800) participated in this study, of whom 728 were infected with *C*. *sinensis*. The overall infection proportion was 28.9%, and the standardized infection proportion was 27.1%. Male (43.6%) experienced higher proportion of infection than female (11.8%) (*χ*^*2*^ = 308.482, *p<*0.01), and the different was significant between people <15 years old (2.2%) and other age groups (38.6%) (*χ*^*2*^ = 297.293, *p<*0.01), between student/children (2.1%) and other occupations (37.6%) (*χ*^*2*^ = 287.804, *p<*0.01), and between illiterate/preschool (20.7%) and other education levels (39.3%) (*χ*^*2*^ = 4.136, *p<*0.05).

Of the 728 residents who were infected with *C*. *sinensis*, 590 (81.0%) were male. The age of the infected persons ranged from 3 to 88 years, with an average age of 50 years and a median age of 51 years (interquartile range: 41–60). Most of the infected persons (97.8%) were adults aged over 15 years old and 485 (66.6%) infected persons were between 30–59 years old. 628 (86.3%) were farmers and the number of infected persons with education level of junior high school was 434 (59.6%).Additionally, 2 (0.3%) were co-infected with trichurids (Table 1).

**Table 1:** Demographic and Infection Intensity Characteristics of the Screened Subjects in Binyang County.

Feature	Screening objects	No.infection (%)	Geometric mean epg among infection persons (SD)	Infection intensity (%)		
				Mild	Moderate	Heavy
**Overall**	2521	728(28.9)	381(7)	482(66.2)	207(28.4)	39(5.4)
**Co-infection**						
With trichurid	728	2(0.3)				
**Gender**						
Male	1353	590(43.6)	484(7)	369(62.5)	184(31.2)	37(6.3)
Female	1168	138(11.8)	137(6)	113(81.9)	23(16.7)	2(1.4)
**Age group**						
<15	625	14(2.2)	72(4)	13(92.9)	1(7.1)	0(0.0)
15–29	158	34(21.5)	266(7)	23(67.6)	11(32.4)	0(0.0)
30–44	464	189(40.7)	379(7)	125(66.1)	56(29.6)	8(4.2)
45–59	655	296(45.2)	466(7)	184(62.2)	90(30.4)	22(7.4)
>59	565	192(34.0)	350(7)	134(69.8)	49(25.5)	9(4.7)
**Ocupation**						
Student and children	621	13(2.1)	78(4)	12(92.3)	1(7.7)	0(0.0)
Farmer	1651	628(38.0)	395(7)	412(65.6)	182(29.0)	34(5.4)
Others	249	87(34.9)	375(7)	58(66.7)	24(27.6)	5(5.7)
**Education**						
Illiteracy and preschool	29	6(20.7)	135(3)	6(100.0)	0(0.0)	0(0.0)
Primary school	527	175(33.2)	401(7)	118(67.4)	46(26.3)	11(6.3)
Junior high school	1050	434(41.3)	419(7)	274(63.1)	136(31.3)	24(5.5)
Senior high school	128	56(43.8)	420(6)	38(67.9)	15(26.8)	3(5.4)
University	68	31(45.6)	307(6)	22(71.0)	8(25.8)	1(3.2)

Note: *indicates significant differences in the infection proportion.

# indicates significant differences between mild-intensity infection and high-intensity infection (moderate and heavy infection).

### Infection intensity

Among the 728 persons infected with *C*. *sinensis*, the geometric mean EPG was 381, and the percentages of mild, moderate and heavy infections were 66.2%, 28.4% and 5.4%, respectively. A significant difference in infection intensity was identified between males and females (*χ*^*2*^ = 19.466, *p<*0.01). Males (37.5%) developed a higher percentage of moderate and heavy infections than females (18.1%) (p<0.01). The percentage of moderate and heavy infections increased from 7.1% in <15-year-olds to 37.8% in 45-59-year-olds and then decreased in >59-year-olds (30.2%) (Table 1).

**Fig 2:**
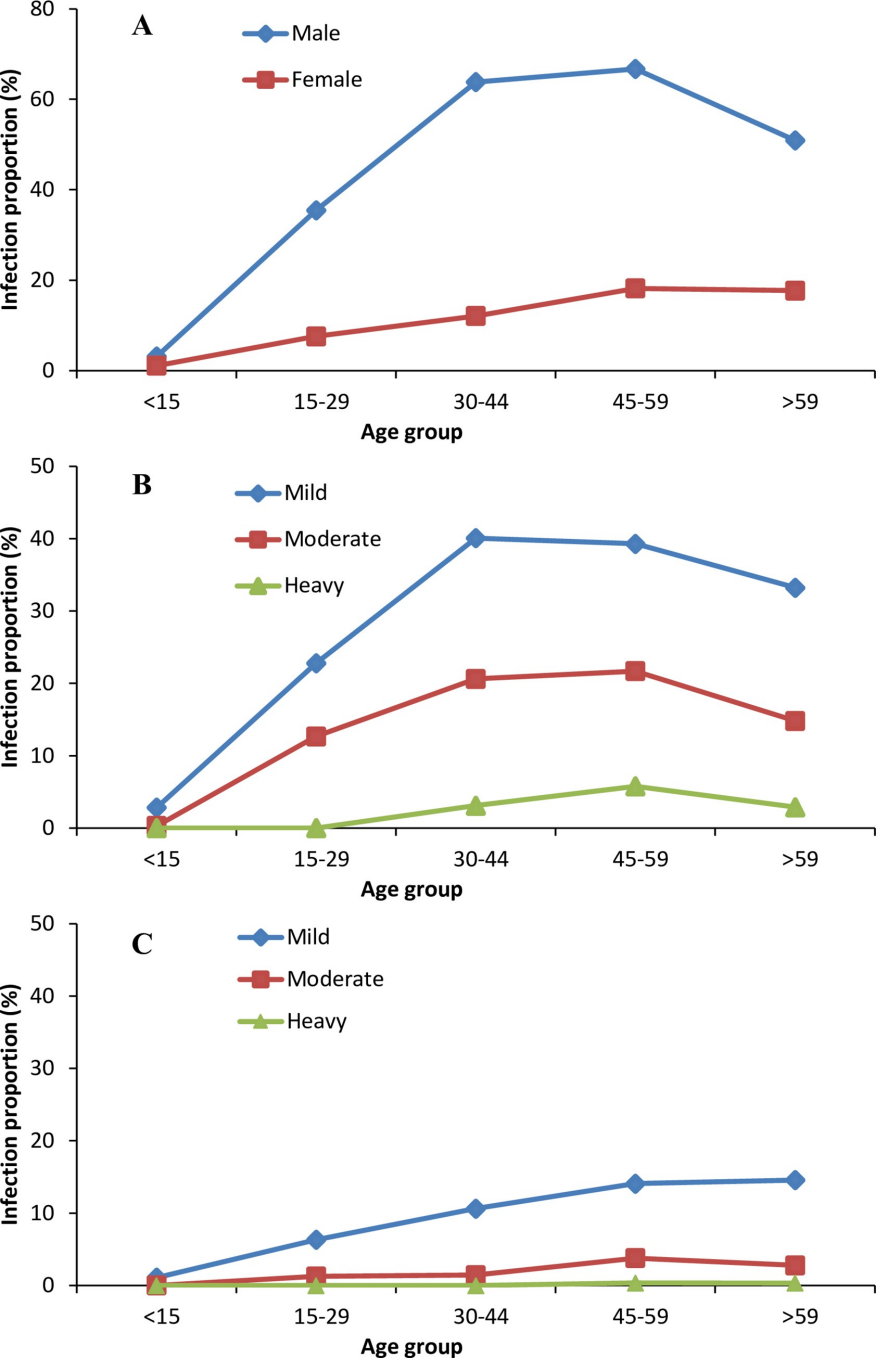
Infection Proportion of Clonorchiasis in Binyang County, Guangxi Autonomous Region, China. **A**. Infection proportion of clonorchiasis in different age groups by sex. **B**. Infection proportion of clonorchiasis for males by age group and infection intensity (mild, moderate and heavy). **C**. Infection proportion of clonorchiasis for females by age group and infection intensity (mild, moderate and heavy).

The proportion of infected males was higher than the proportion of infected females in all age groups, except for children under 15 years old (Fig 2A). In all age groups except for children under 15 years old, the proportion of mild infection was higher than the proportion of moderate and heavy infections both in males and females (Fig 2B and 2C). The infection proportions for all three types of infection intensity increased with age in those aged from <15 years old to 30–59 years old and then began to decrease in those >59 years old (Fig 2).

### Risk behaviour among different groups

Among the 728 infected persons, 538 (73.9%) participated in the questionnaire, of whom 509 (94.6%) reported eating raw fish. Males (96.2%) had a higher rate of raw fish consumption than females (86.8%) (*χ*^*2*^ = 13.055, *p<*0.01). Among the 509 persons who reported eating raw fish, 302 (59.3%) had eaten raw fish for >10 years, 131 (25.7%) ate raw fish ≥12 times a year, and 291 (57.2%) ate ≥20 slices every time they ate raw fish. Four hundred ninety-four (97.1%) did not take anti-parasitics after eating raw fish, and 432 (84.9%) reported eating raw fish at home (Table 2).

**Table 2:** Raw Fish Consumption Behaviour of Different Groups of Infected Persons in Binyang County.

Feature	Eat raw fish	Years of raw fish consumption (n, %)			Frequency of raw fish consumption(times in the last year) (n, %)			Quantity of raw fish consumption(slices per serving) (n, %)			Use of anti-parasitic after eating raw fish (n, %)		Location of raw fish consumption (n, %)	
		≤5	6–10	>10	≤11	12–50	>50	≤9	10–19	≥20	No	Yes	Restaurant	Home
**Overall (N = 538)**	509(94.6)	118(23.2)	89(17.5)	302(59.3)	378(74.3)	107(21.0)	24(4.7)	95(18.7)	123(24.2)	291(57.2)	494(97.1)	15(2.9)	77(15.1)	432(84.9)
**Gender**														
Male (N = 447)	430(96.2)	91(21.2)	69(16.0)	270(62.8)	303(70.5)	103(24.0)	24(5.6)	51(11.9)	108(25.1)	271(63.0)	415(96.5)	15(3.5)	74(17.2)	356(82.8)
Female (N = 91)	79(86.8)	27(34.2)	20(25.3)	32(40.5)	75(94.9)	4(5.1)	0(0.0)	44(55.7)	15(19.0)	20(25.3)	79(100.0)	0(0.0)	3(3.8)	76(96.2)
**Age group**														
0–29 (N = 24)	19(79.2)	14(73.7)	4(21.1)	1(5.3)	17(89.5)	2(10.5)	0(0.0)	6(31.6)	8(42.1)	5(26.3)	19(100.0)	0(0.0)	2(10.5)	17(89.5)
30–44 (N = 140)	136(97.1)	45(33.1)	36(26.5)	55(40.4)	108(79.4)	21(15.4)	7(5.1)	25(18.4)	41(30.1)	70(51.5)	131(96.3)	5(3.7)	34(25.0)	102(75.0)
45–59 (N = 227)	218(96.0)	35(16.1)	31(14.2)	152(69.7)	150(68.8)	54(24.8)	14(6.4)	37(17.0)	49(22.5)	132(60.6)	213(97.7)	5(2.3)	31(14.2)	187(85.8)
>60 (N = 147)	136(92.5)	24(17.6)	18(13.2)	94(69.1)	103(75.7)	30(22.1)	3(2.2)	27(19.9)	25(18.4)	84(61.8)	131(96.3)	5(3.7)	10(7.4)	126(92.6)
**Occupation**														
Farmer (N = 461)	439(95.2)	87(19.8)	76(17.3)	276(62.9)	322(73.3)	96(21.9)	21(4.8)	78(17.8)	100(22.8)	261(59.5)	426(97.0)	13(3.0)	68(15.5)	371(84.5)
Others (N = 77)	70(90.9)	31(44.3)	13(18.6)	26(37.1)	56(80.0)	11(15.7)	3(4.3)	17(24.3)	23(32.9)	30(42.9)	68(97.1)	2(2.9)	9(12.9)	61(87.1)
**Education**														
Primary school or below (N = 197)	180(91.4)	34(18.9)	30(16.7)	116(64.4)	128(71.1)	43(23.9)	9(5.0)	40(22.2)	32(17.8)	108(60.0)	174(96.7)	6(3.3)	24(13.3)	156(86.7)
Junior high school (N = 266)	257(96.6)	56(21.8)	48(18.7)	153(59.5)	192(74.7)	53(20.6)	12(4.7)	42(16.3)	68(26.5)	147(57.2)	251(97.7)	6(2.3)	36(14.0)	221(86.0)
Senior high school or above (N = 75)	72(96.0)	28(38.9)	11(15.3)	33(45.8)	58(80.6)	11(15.3)	3(4.2)	13(18.1)	23(31.9)	36(50.0)	69(95.8)	3(4.2)	17(23.6)	55(76.4)
**Previous diagnosis with clonorchiasis**														
Yes (N = 57)	57(100.0)	5(8.8)	7(12.3)	45(78.9)	35(61.4)	17(29.8)	5(8.8)	7(12.3)	18(31.6)	32(56.1)	52(91.2)	5(8.8)	11(19.3)	46(80.7)
No (N = 481)	452(94.0)	113(25.0)	82(18.1)	257(56.9)	343(75.9)	90(19.9)	19(4.2)	88(19.5)	105(23.2)	259(57.3)	442(97.8)	10(2.2)	66(14.6)	386(85.4)
**Previously use of anti-parasitic**														
Yes (N = 50)	50(100.0)	4(8.0)	7(14.0)	39(78.0)	30(60.0)	15(30.0)	5(10.0)	5(10.0)	16(32.0)	29(58.0)	46(92.0)	4(8.0)	10(20.0)	40(80.0)
No (N = 488)	459(94.1)	114(24.8)	82(17.9)	263(57.3)	348(75.8)	92(20.0)	19(4.1)	90(19.6)	107(23.3)	262(57.1)	448(97.6)	11(2.4)	67(14.6)	392(85.4)
**Knowledge of the transmission route of clonorchiasis**														
Yes (N = 308)	304(98.7)	70(23.0)	50(16.4)	184(60.5)	220(72.4)	65(21.4)	19(6.3)	45(14.8)	76(25.0)	183(60.2)	291(95.7)	13(4.3)	46(15.1)	258(84.9)
No (N = 230)	205(89.1)	48(23.4)	39(19.0)	118(57.6)	158(77.1)	42(20.5)	5(2.4)	50(24.4)	47(22.9)	108(52.7)	203(99.0)	2(1.0)	31(15.1)	174(84.9)
**Knowledge of potential to cause cancer**														
Yes (N = 57)	55(96.5)	12(21.8)	5(9.1)	38(69.1)	39(70.9)	15(27.3)	1(1.8)	9(16.4)	13(23.6)	33(60.0)	52(94.5)	3(5.5)	6(10.9)	49(89.1)
No (N = 481)	454(94.4)	106(23.3)	84(18.5)	264(58.1)	339(74.7)	92(20.3)	23(5.1)	86(18.9)	110(24.2)	258(56.8)	442(97.4)	12(2.6)	71(15.6)	383(84.4)

Among the 509 persons who reported eating raw fish, higher percentages of males than females had eaten raw fish for >10 years (62.8% *vs* 40.5%, *χ*^*2*^ = 13.735, *p<*0.01), ate raw fish ≥12 times a year (29.6% *vs* 5.1%, *χ*^*2*^ = 20.911, *p<*0.01) and ate ≥20 slices every time they ate raw fish (63.0% *vs* 25.3%, *χ*^*2*^ = 38.753, *p<*0.01). Additionally, a higher percentage of males (17.2%) than females (3.8%) reported eating raw fish at restaurants (*χ*^*2*^ = 9.350, *p<*0.01). Higher percentages of persons who had been previously diagnosed with clonorchiasis than those who had not been previously diagnosed reported eating raw fish for >10 years (78.9% *vs* 56.9%, *χ*^*2*^ = 10.235, *p<*0.01) and eating raw fish ≥12 times a year (38.6% *vs* 24.1%, *χ*^*2*^ = 5.554, *p* = 0.018). Furthermore, higher percentages of those who had previously taken anti-parasitics than those who had not taken anti-parasitics reported having eaten raw fish for >10 years (78.0% *vs* 57.3%, *χ*^*2*^ = 8.008, *p<*0.01) and eating raw fish ≥12 times a year (40.0% *vs* 24.1%, *χ*^*2*^ = 5.902, *p* = 0.015). No significant differences in raw fish consumption behaviour were identified between those with different knowledge of clonorchiasis (knowledge of the transmission route of clonorchiasis and that it causes cancer) (Table 2).

### Risk factors of high infection intensity

Among the 509 persons who reported eating raw fish, 195 (38.3%) were classified as having moderate or heavy infection intensity. The percentage of moderate and heavy intensity of infection had increased from 32.8% among people consuming raw fish 0–11 times per year, to 51.4% for consuming raw fish 12–50 times per year, and to 66.7% for consuming raw fish >50 times per year. The univariate analysis revealed that the risk factors of moderate and heavy infection intensity included eating raw fish for > 10 years (crude odds ratio [cOR] = 1.94, 95%CI = 1.24~3.10), eating raw fish 12–50 times a year (cOR = 2.17, 95%CI = 1.40~3.36), eating raw fish >50 times a year (cOR = 4.10, 95%CI = 1.75~10.35) and eating ≥ 20 slices of raw fish per serving (cOR = 2.39, 95%CI = 1.45~4.05). However, the multivariate logistic regression analysis showed that only two subgroups had higher risks of moderate and heavy infection intensity: those who ate raw fish 12–50 times a year (adjusted odds ratio [aOR] = 1.74, 95%CI = 1.09~2.80) and those who ate raw fish >50 times a year (aOR = 2.89, 95%CI = 1.20~7.50) (Table 3).

**Table 3:** Risk Factors of High Infection Intensity (Moderate and Heavy Infections) Based on Multivariate Logistic Regression.

Features	Total	Moderate or heavy intensity of infection (n, %)	cOR^*^ *(95%CI)*	aOR^**^ *(95%CI)*
**Years of raw fish consumption**				
0–5	118	34(28.8)	Ref	Ref
6–10	89	28(31.5)	1.13(0.62–2.06)	0.95(0.50–1.76)
>10	302	133(44.0)	1.94(1.24–3.10)	1.21(0.71–2.07)
**Frequency of raw fish consumption**				
0–11 times per year	378	124(32.8)	Ref	Ref
12–50 times per year	107	55(51.4)	2.17(1.40–3.36)	1.74(1.09–2.80) ^#^
>50 times per year	24	16(66.7)	4.10(1.75–10.35)	2.89(1.20–7.50) ^#^
**Quantity of raw fish consumption**				
0–9	95	25(26.3)	Ref	Ref
10–19	123	36(29.3)	1.16(0.64–2.13)	1.01(0.55–1.89)
≥20	291	134(46.0)	2.39(1.45–4.05)	1.66(0.94–2.99)
**Location of raw fish consumption**				
Restaurant and others	77	34(44.2)	Ref	Ref
Home	432	161(37.3)	0.75(0.46–1.23)	0.78(0.47–1.31)
**Use of anti-parasitic after eating raw fish**				
No	494	188(38.1)	Ref	Ref
Occasionally	15	7(46.7)	1.42(0.49–4.03)	1.24(0.41–3.67)
**Previous diagnosis with clonorchiasis before**				
Yes	57	23(40.4)	Ref	Ref
No	481	172(35.8)	0.91(0.52–1.61)	1.15(0.63–2.15)
**Knowledge of the infection route of clonorchiasis**				
Yes	308	127(41.2)	Ref	Ref
No	230	71(30.9)	0.72(0.49–1.03)	0.75(0.50–1.12)
**Knowledge of the potential to cause cancer**				
Yes	57	23(40.4)	Ref	Ref
No	481	175(36.4)	0.92(0.52–1.65)	1.07(0.58–2.00)

* cOR: crude odds ratio

** aOR: adjusted odds ratio

# Statistical differences identified.

## Discussion

In this study, by analysing cross-sectional data from an endemic area in China, we found that the infection proportion of clonorchiasis was high and that a large proportion of infected persons had moderate and heavy intensity infections. People aged 30–59 years had a higher infection proportion and a higher percentage of moderate and heavy infections than those in the other age groups. Males experienced a higher percentage of moderate and heavy intensity infections than females, which might be attributed to the longer duration, higher frequency and greater quantity of their raw fish consumptions. Additionally, people who had been previously diagnosed with clonorchiasis or had previously taken anti-parasitics had longer durations and higher frequencies of raw fish consumptions, while no differences were identified among people with different knowledge of clonorchiasis. Furthermore, a high frequency of raw fish consumption was a risk factor for a high intensity of infection.

The distribution characteristics of infection intensity and infection proportion among the sexes and age groups except among children that were identified in this study were mainly related to living customs, i.e., raw fish consumption. Raw fish are often consumed in social gathering or at restaurants, and offering raw fish to guests is deemed a hospitable gesture; males have more opportunities to participate in these practices [8,28,29]. Moreover, raw fish are often enjoyed with alcoholic beverages, which is more common among males than females, except for among children [5,30]. Furthermore, adult worms can survive in the body for decades. Consequently, adult males’ exposure and, consequently, their worm load are higher, which leads to higher infection intensity and higher prevalence. The decline in the overall prevalence of infection and the three types of infection intensity in those older than 60 is probably due to early death caused by clonorchiasis-related complications [5,15]. Additionally, elderly individuals seek medical services more frequently due to clonorchiasis-related complications or unrelated diseases and then accept diagnosis and treatment [5].

High frequency of raw fish consumption was associated with high intensity of infection, which was consistent with the results from a study conducted in one community in Shunde district, Guangdong province, P.R. China [25]. However, different from previous research that direct compared the geometric means of EPG (GMEPG) for different variable group, we re-classified the quantitative variable (EPG) into categorical variable, and a multivariable analysis was used to explore the relationship between raw fish consumption and high intensity of infection.

At present, the management of clonorchiasis is focused on morbidity control with praziquantel [31]. In moderately endemic areas (prevalence rate: 20%-40%) [32], such as the area where our study site is located, yearly administration of praziquantel under selective chemotherapy resulted in a substantial decrease in the prevalence and intensity of infection within 3 years [32]. However, the sustainability of long-term achievements is challenging, as re-infection cannot be avoided using chemotherapy alone, especially in older age groups [25,30,33]. Information, education and communication (IEC) is usually combined with chemotherapy to enhance sustainability [6,34]. However, according to our study, no significant difference in raw fish consumption behaviour was identified among people with different knowledge of clonorchiasis. Hence, other control measures should be implemented in endemic areas. Currently, in local endemic areas of China, particularly in village regions, toilets are built directly above or beside fish ponds, and unprocessed faeces can contaminate the water, which increases *C*. *sinensis* infection in snails and freshwater fish [35]. Sanitary toilets with a harmless processing design (sanitary toilets own extra pools to store and precipitate feces during which the marsh gas is produced and the parasite eggs are killed), which have been previously implemented in one epidemic area in China with promising effects [35], should be tried in other endemic areas. Moreover, residents in epidemic areas find it difficult to change their raw fish consumption habits. Therefore, more attention should be paid to the safety of freshwater fish. The infection rates and distribution of freshwater fish should be investigated in endemic areas, and infected ponds should be placed under surveillance. Additionally, reservoir hosts such as cats or dogs, found to harbour *C*. *sinensis* at a prevalence of as high as 65% can contribute to contamination of fresh water bodies [36]. Therefore, health education should be implemented in endemic areas to intervene tradition of feeding cats or dogs with raw fish or raw fish viscera. Currently, the direct compression or artificial digestion of fish followed by detection under a microscope is used to examine *C*. *sinensis* metacercaria in freshwater fish, which is time consuming, labour-intensive and can allow *C*. *sinensis* eggs to be easily confused with other parasite eggs [37–39]. Meanwhile, PCR-based molecular biology techniques are expensive [37]; thus, rapid, convenient, inexpensive and accurate detection methods are urgently needed. Moreover, a notable avenue of vaccine research is the vaccination of the second intermediate host, i.e., freshwater fish, with feed probiotics. Indeed, an oral vaccine based on *Bacillus subtilis* expressing enolase is being tested in freshwater fish [6,40].

Two limitations exists in this study. Firstly, among the 728 persons who were infected with *C*. *sinensis*, only 538 (73.9%) participated in our questionnaire investigation. Therefore, the results might have been influenced. However, we compared the basic demographic information (sex and age) between these 538 subjects and the 190 non-participating persons, and no significant difference was identified. Thus, we believe this had a limited effect on the results. Secondly, the diagnosis method (Kato-Katz method) used in this study is low sensitivity, especially for detection of low-intensity infection. Additionally, using this method to distinguish C. sinensis eggs from other liver flukes (e.g., *O viverrini* and *Opisthorchis felineus*) and minute intestinal flukes is very difficult.

Based on our results, the overall infection proportion and intensity of clonorchiasis were high at the study site, with males experiencing higher intensity of infection than females. People 30–59 years old had a higher burden of clonorchiasis than those in the other age groups. In addition, males showed a higher risk of raw fish consumption than females, and no difference in raw fish consumption behaviour was noted among people with different knowledge of clonorchiasis. A high frequency of raw fish consumption was associated with a high intensity of infection. More measures focused on contaminated faeces and intermediates, such as the reconstruction of toilets and the examination of freshwater fish, should be implemented along with chemotherapy and IEC in local endemic areas.

## Supporting information

S1 Database Demographic, infection information of research subjects. (XLSX)
(XLSX)
